# Preoperative three-dimensional lung volumetry predicts respiratory complications in patients undergoing major liver resection for colorectal metastases

**DOI:** 10.1038/s41598-024-61386-8

**Published:** 2024-05-08

**Authors:** Suzan Elmaagacli, Christoph Thiele, Franziska Meister, Philipp Menne, Daniel Truhn, Steven W. M. Olde Damink, Johannes Bickenbach, Ulf Neumann, Sven Arke Lang, Florian Vondran, Iakovos Amygdalos

**Affiliations:** 1 Department of General, Visceral, Pediatric, and Transplantation Surgery, University Hospital RWTH Aachen, Pauwelsstraße 30, 52074 Aachen, Germany; 2 Department of Operative Intensive Care and Intermediate Care, University Hospital RWTH Aachen, Aachen, Germany; 3 Department of Diagnostic and Interventional Radiology, University Hospital RWTH Aachen, Aachen, Germany; 4 Department of Surgery, Maastricht University Medical Center+, Maastricht, The Netherlands

**Keywords:** Colorectal liver metastases, Hepatobiliary, Surgery, Lung volumetry, Respiratory complications, Outcomes research, Colorectal cancer, Liver cancer

## Abstract

Colorectal liver metastases (CRLM) are the predominant factor limiting survival in patients with colorectal cancer and liver resection with complete tumor removal is the best treatment option for these patients. This study examines the predictive ability of three-dimensional lung volumetry (3DLV) based on preoperative computerized tomography (CT), to predict postoperative pulmonary complications in patients undergoing major liver resection for CRLM. Patients undergoing major curative liver resection for CRLM between 2010 and 2021 with a preoperative CT scan of the thorax within 6 weeks of surgery, were included. Total lung volume (TLV) was calculated using volumetry software 3D-Slicer version 4.11.20210226 including Chest Imaging Platform extension (http://www.slicer.org). The area under the curve (AUC) of a receiver-operating characteristic analysis was used to define a cut-off value of TLV, for predicting the occurrence of postoperative respiratory complications. Differences between patients with TLV below and above the cut-off were examined with Chi-square or Fisher’s exact test and Mann–Whitney U tests and logistic regression was used to determine independent risk factors for the development of respiratory complications. A total of 123 patients were included, of which 35 (29%) developed respiratory complications. A predictive ability of TLV regarding respiratory complications was shown (AUC 0.62, *p* = 0.036) and a cut-off value of 4500 cm^3^ was defined. Patients with TLV < 4500 cm^3^ were shown to suffer from significantly higher rates of respiratory complications (44% vs. 21%, *p* = 0.007) compared to the rest. Logistic regression analysis identified TLV < 4500 cm^3^ as an independent predictor for the occurrence of respiratory complications (odds ratio 3.777, 95% confidence intervals 1.488–9.588, *p* = 0.005). Preoperative 3DLV is a viable technique for prediction of postoperative pulmonary complications in patients undergoing major liver resection for CRLM. More studies in larger cohorts are necessary to further evaluate this technique.

## Introduction

Colorectal cancer (CRC) is the third most common cancer worldwide and colorectal liver metastases (CRLM) can affect up to 80% of patients with CRC^1,2^. The best option for cure or long-term survival in patients with CRLM is liver resection with complete tumor removal. This may be carried out directly in cases of primarily resectable disease (approximately 30%), or after tumor downsizing through systemic chemotherapy^3,4^.

Patients with CRLM often require multimodal treatment approaches, which may include perioperative chemotherapy, local ablative techniques, portal vein embolization (PVE), and staged resections, including Associating Liver Partition and Portal vein ligation for Staged hepatectomy (ALPPS)^1,3,5^. Patients requiring major liver resection for CRLM are at increased risk of perioperative morbidity and mortality, despite technical advances and high experience of specialized centers^6^. Previous attempts have been made to develop scores and algorithms, which can identify patients at risk for high morbidity and mortality following liver surgery^7–9^. Postoperative pulmonary complications, in particular, are a major cause of mortality and morbidity and accountable for longer intensive care unit (ICU) stays after liver surgery^10^. Furthermore, due to an increase in overall life expectancy, more elderly patients undergo major liver resection^11^, which is associated with increased rate of pulmonary complications^10^. Therefore, the ability to identify patients at risk for postoperative pulmonary complications is of great clinical relevance.

Three-dimensional lung volumetry (3DLV) based on computerized tomography (CT) is a novel technique, which can be performed on routine staging CT scans of the thorax. It provides a technically stable estimation of lung volume^12^, which is well-correlated with spirometric measurements of total lung capacity^13^. The capability of 3DLV to reliably predict postoperative pulmonary function has been previously established in thoracic surgery^13–16^. In a novel approach, the aim of this study was to examine the ability of preoperative 3DLV to predict postoperative respiratory complications, postoperative overall morbidity, and length of ICU- and hospital stay after major curative liver resection for CRLM.

## Methods

### Patient cohort and inclusion criteria

Consecutive adult patients undergoing elective major liver resection for CRLM at the University Hospital RWTH Aachen (UH-RWTH) between 2010 and 2021, with a preoperative CT of the thorax within 6 weeks of operation, were eligible for inclusion in this retrospective study. Liver resections were described according to the Brisbane classification^17^ and defined as major, when ≥ 3 segments were resected. Patients were excluded, if they underwent resections of hepatic metastatic recurrence, or exploration without resection. Patients with inadequate quality of the thorax CT (for example, where lungs were not completely captured) or evidence of active lung disease, were also excluded.

The primary endpoint of the study was the incidence of postoperative respiratory complications within 90 days of operation, defined as the occurrence of any of the following: respiratory failure, pulmonary embolism, pneumonia, pleural empyema, pleural effusion requiring thoracentesis and pneumothorax necessitating thoracic drainage. Further perioperative outcomes, such as cumulative morbidity defined by the Comprehensive Complication Index (CCI)^18^ and major complications according to the Clavien-Dindo score (CD ≥ 3)^19^, as well as length of intensive care unit (ICU)- and hospital stay were examined as secondary endpoints.

### Data collection

Clinical data were recovered from a prospective institutional database and analyzed retrospectively. These included demographic information such as body mass index (BMI) and American Society of Anesthesiology (ASA) score, radio-oncological staging, and treatment strategies. Primary tumors located in the cecum to transverse colon were categorized as right-sided, whereas tumors located from the splenic flexure to rectum as left-sided. Histological staging of the primary tumor was documented according to the Union for International Cancer Control (UICC) tumor/lymph node/metastasis system (TNM). Kirsten rat sarcoma viral oncogene homolog (*KRAS*) mutation status was obtained from histological analysis of either the primary tumor or CRLM (with mutated status overriding wildtype in any configuration). Synchronous metastases were defined as those being diagnosed simultaneously or within 3 months of the primary tumor. The number, size, and location of CRLM were obtained from preoperative computerized tomography (CT) and corroborated with liver-protocol magnetic resonance imaging (MRI), where available^9^. Preoperative chemotherapy was defined as any systemic treatment administered within six months before surgery. Radicality of resection (denoted R0 for radical, otherwise R+) was determined by postoperative pathological analysis. Postoperative extubation point was recorded as either in the operating room or on ICU^9^. Respiratory complications were diagnosed commonly by X-ray or CT.

**Figure 1:**
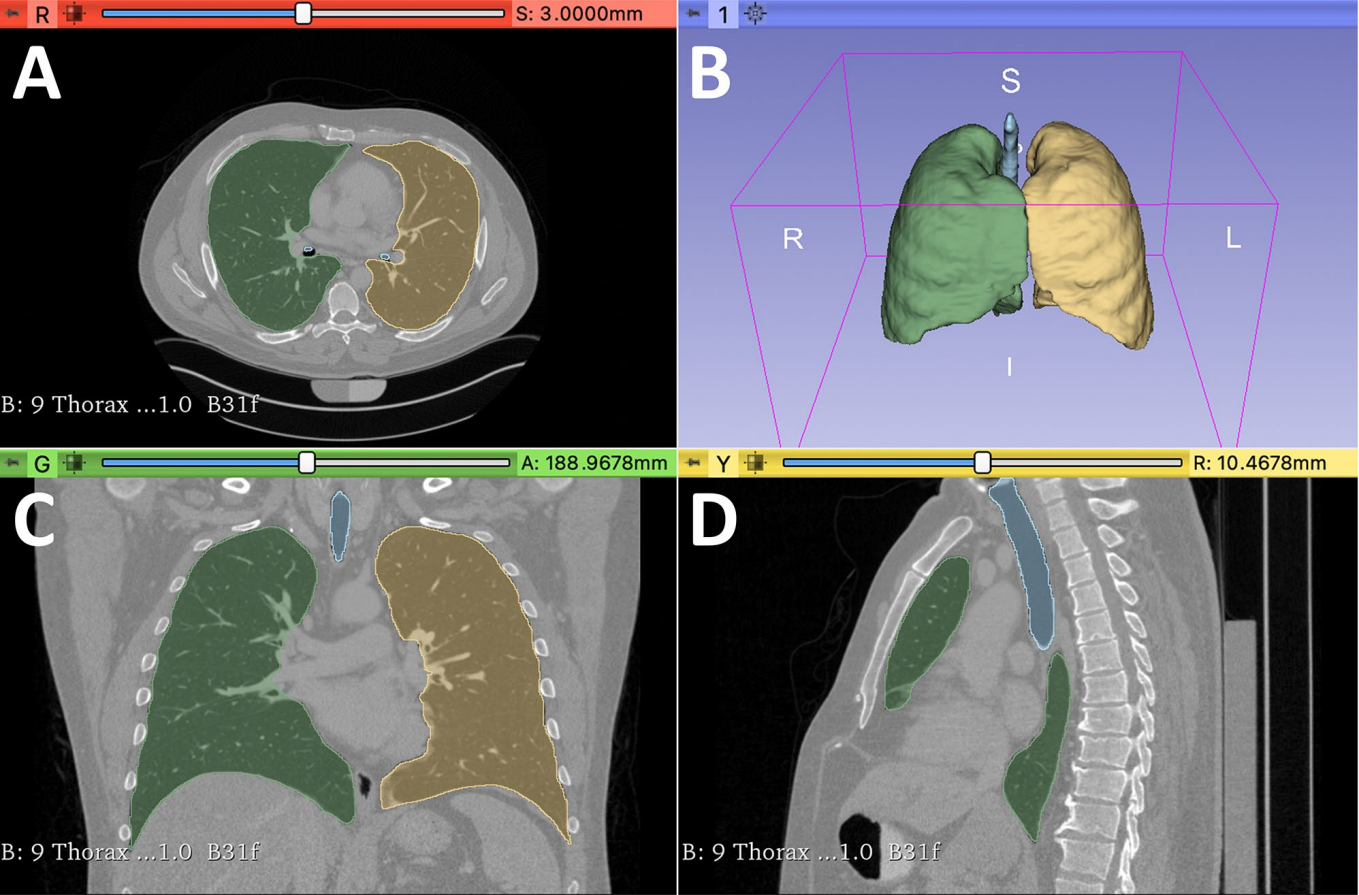
Three-dimensional computer-tomography-based lung volumetry of a male patient. (**A**) axial view; (**B**) Three-dimensional model of the respiratory system; (**C**) coronal view; (**D**) sagittal view. Green shading: right lung; Yellow shading: left lung; Blue shading: trachea with main bronchi; Total lung volume calculated as the sum of the right and left lung volumes.

Routine preoperative staging CT scans of the thorax, within 6 weeks of liver resection, were used for 3DLV measurements. Suitable scans were identified using electronic patient records and the hospital Picture Archiving and Communication System (PACS). Anonymized Digital Imaging and Communications in Medicine (DICOM) image series were exported for analysis and organized according to an identification number, matched to the patient data from our database. Exported series were loaded into the software 3D-Slicer version 4.11.20210226 including the Chest Imaging Platform extension (http://www.slicer.org). Axial, coronal, and sagittal views were used for the analysis. Manual marking was used to identify lung parenchyma and mapping of lung volume was carried out automatically. For this, lung volume was quantified using a predefined Hounsfield unit (HU) range of − 1000 HU to − 200 HU. Trachea and bronchial system volume was automatically removed by the software, whereas pneumatic intestinal loops and any errors from the automatic process were manually corrected. Figure 1 shows an example of a 3DLV.

### Operative technique

Operative technique adhered to common clinical standards for liver resection^9^. Intraoperative sonography confirmed preoperative imaging findings and ruled out new or undetected lesions. The Cavitron Ultrasonic Surgical Aspirator (CUSA^®^, Integra LifeSciences, Plainsboro NJ, USA) was used for parenchymal transection in open surgery, with clipping or ligation of vascular and biliary structures. In laparoscopic cases, either the THUNDERBEAT (Olympus K.K., Tokyo, Japan), HARMONIC ACE^®^ (Ethicon Inc. Somerville, NJ, USA) or laparoscopic CUSA^®^ (Integra LifeSciences, Plainsboro NJ, USA) devices were combined with ECHELON™ vascular staplers (Ethicon, Somerville, New Jersey, USA) or Weck^®^ Hem-o-lok^®^ polymer clips (Teleflex Inc., Pennsylvania, USA). Intermittent Pringle maneuvers were carried out as needed. Anatomical or parenchyma-sparing resections were carried out according to general patient condition, preoperative liver function tests, and limiting factors, such as macrovascular invasion. Radicality of tumor resection was controlled through frozen section. Anesthesiologic management aimed for a low central venous pressure (CVP) during the resection phase.

### Statistical analysis

The Kolmogorov–Smirnov test was used to check data for normal distribution. Data were described as medians and interquartile range (IQR, given as 25th–75th percentiles) for non-normally distributed continuous variables, or absolute and relative frequencies for categorical and ordinal variables. To examine the predictive capability of 3DLV regarding respiratory complications, the area under the curve (AUC) of the receiver operating characteristic (ROC) analysis was calculated. Group comparisons were performed using the Chi-square test or Fisher’s exact test and the Mann–Whitney-U test. Uni- and multivariable binary logistic regression was used to determine independent risk factors for the development of respiratory complications and corresponding odds ratios (OR) were given with 95% confidence intervals (CI). Only *p*-values < 0.05 were considered statistically significant. Statistical analysis was performed using SPSS Statistics version 28.0.1.0 (IBM Corp., Armonk, NY, USA). Analysis was carried out using the whole cohort and repeated for subgroups of interest. These were: age above 60 years, BMI classification according to the World Health Organization (WHO)^20^, and patients undergoing ALPPS. The latter were examined separately, because of the increased complexity of the procedure and associated increased morbidity and mortality^21^.

### Ethical approval

The study was conducted under the ethical approval of the Institutional Review Board of the RWTH Aachen University (EK-001/21) and in accordance with the current version of the Declaration of Helsinki, the Declaration of Istanbul, and good clinical practice guidelines (ICHGCP). Informed consent was waived due to the retrospective study design and collection of readily available clinical data.

## Results

### Patient characteristics

**Figure 2:**
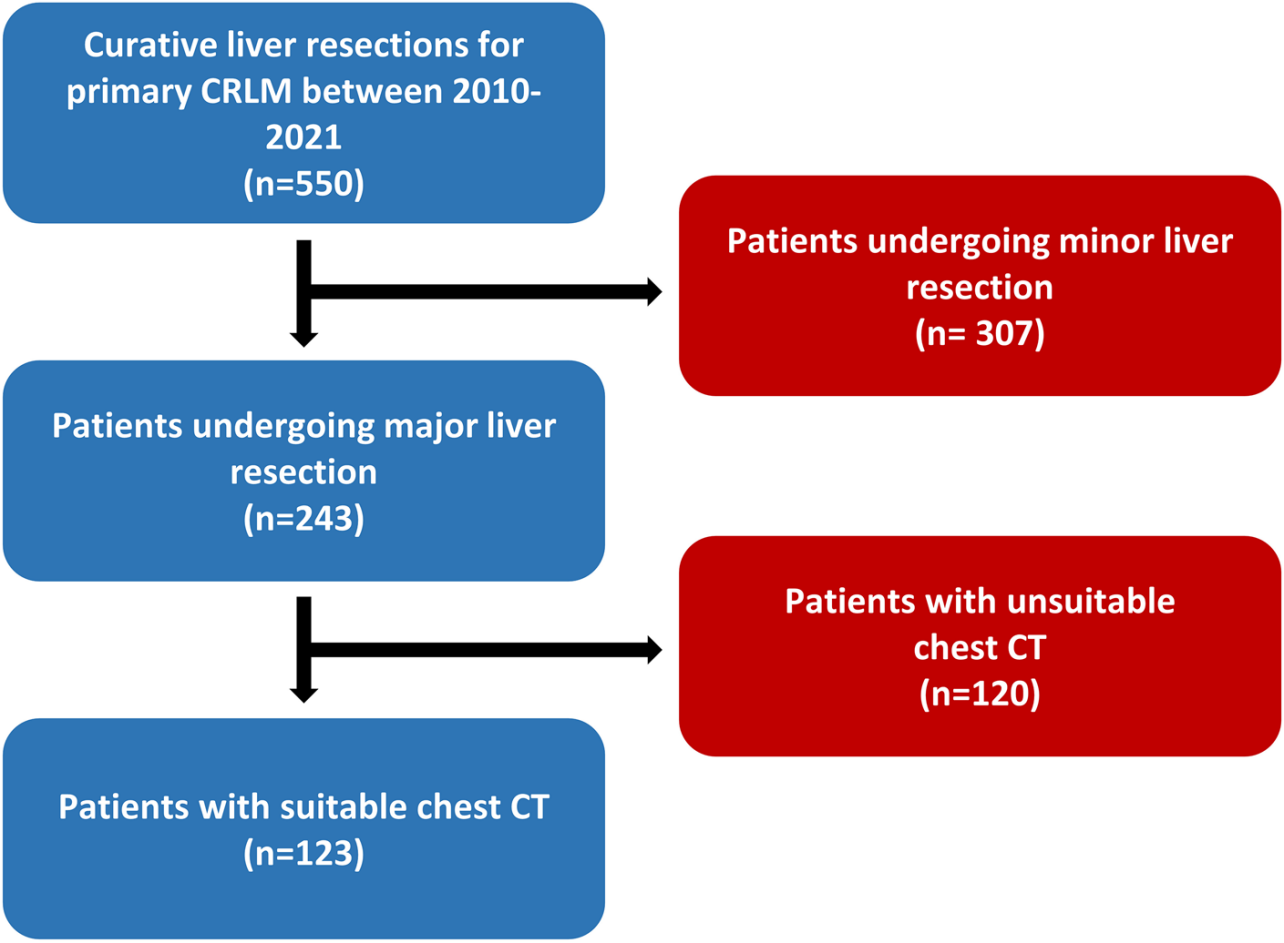
Flowchart of inclusion and exclusion criteria, leading to the final study population. Abbreviations: CRLM, colorectal liver metastases; CT, Computerised Tomography.

Between 2010 and 2021, 550 patients underwent curative liver resections for CRLM at the UH-RWTH. Of these, 243 received major resections, 124 of which had a CT scan of the thorax within 6 weeks before surgery. One patient was excluded, because the CT scan did not include the whole lung, resulting in a final cohort of 123 patients (Fig. 2). The median age of the study cohort was 62 years, and 59% of patients had an ASA score of ≥ III. Thirty-five (29%) patients developed respiratory complications, as there were: respiratory failure (11.4%), pulmonary embolism (8.6%), pneumonia (14.3%), pleural empyema (2.9%), pleural effusion requiring thoracentesis (74.3%) and pneumothorax necessitating thoracic drainage (17.1%) (sum is more 100% due to the fact that some patients had more than one complication. All patients with respiratory failure or pulmonary embolism required ICU or intermediate care (IMC) admission, and 100% of patients with pleural effusion or pneumothoraces underwent thoracentesis. The median TLV of the cohort was 4945 cm^3^, with a minimum TLV of 2492 cm^3^ and a maximum TLV of 8444 cm^3^. Patient characteristics of the whole study population are given in Table 1 and postoperative outcomes are described in Table 2.

**Table 1:** Preoperative patient characteristics for the whole study population and groups with total lung volume above and below the cut-off value of 4500 cm^3^.

Variables	All patients (n = 123)	TLV < 4500 cm^3^ (n = 41)	TLV ≥ 4500 cm^3^ (n = 82)	*p*-value
Age (years)	62 [54–68]	61 [52–69]	62 [56–67]	0.953
Sex (female)	51 (41%)	30 (73%)	21 (26%)	** < 0.001**
BMI	25 [23–28]	25 [22–28]	25 [23–28]	0.915
Height in cm	174 [167–180]	168 [160–174]	176 [170–182]	** < 0.001**
Weight in kg	77 [67–87]	70 [60–84]	80 [70–90]	**0.018**
ASA score^a^				
ASA I	4 (3%)	1 (2%)	3 (4%)	0.719
ASA II	46 (38%)	15 (37%)	31 (38%)	0.895
ASA III	69 (56%)	25 (61%)	44 (54%)	0.441
ASA IV	4 (3%)	0 (0%)	4 (5%)	0.150
Synchronous metastases	91 (74%)	31 (76%)	60 (73%)	0.771
Right-sided colorectal cancer	32 (26%)	11 (27%)	21 (26%)	0.884
Preoperative chemotherapy^b^	66 (61%) n = 108	19 (53%) n = 36	47 (65%) n = 72	0.209
Operation extent				
Hemihepatectomy	84 (68%)	30 (73%)	54 (66%)	0.411
Extended hemihepatectomy/trisectionectomy	39 (32%)	11 (27%)	28 (34%)	0.411
Operation type				
Anatomical	78 (63%)	25 (61%)	53 (65%)	0.691
Atypical	0 (0%)	0 (0%)	0 (0%)	n/a
Combined anatomical & atypical	31 (25%)	9 (22%)	22 (27%)	0.557
ALPPS	15 (12%)	7 (17%)	8 (10%)	0.242
Operation technique				
Minimally invasive	23 (19%)	10 (24%)	13 (16%)	0.252
Open	100 (81%)	31 (76%)	69 (84%)	0.252
Staged resection	33 (27%)	11 (27%)	22 (27%)	1
Portal vein embolization (yes)	31 (25%)	9 (22%)	22 (27%)	0.557

Values given as median (1st quartile–3rd quartile) or absolute and relative frequencies; Bold values represent significant values.

*ALPPS*, associating liver partition and portal vein ligation for staged hepatectomy, *ASA* American Society of Anesthesiology, *BMI* body mass index, *TLV* total lung volume.

^a^Refers to Meyer Saklad et al.

^b^Preoperative chemotherapy defined as chemotherapy given 6 months before surgery.

**Table 2:** Perioperative outcomes for the whole study population and groups with total lung volume above and below the cut-off value of 4500 cm^3^.

Variables	All patients (n = 123)	TLV < 4500 cm^3^ (n = 41)	TLV ≥ 4500 cm^3^ (n = 82)	*p*-value
Operating time (min)	295 [239–363]	270 [236–359]	313 [239–365]	0.629
Intraoperative RBC units	0 [0–1]	0 [0–2]	0 [0–1]	0.694
Intraoperative platelet units	0 [0–0]	0 [0–0]	0 [0–0]	0.734
Intraoperative FFP units	0 [0–3]	0 [0–2]	0 [0–4]	0.160
Extubation on ICU	49 (40%)	16 (39%)	33 (40%)	0.896
Respiratory complications	35 (29%)	18 (44%)	17 (21%)	**0.007**
CD ≥ 3a^a^	56 (46%)	22 (54%)	34 (42%)	0.200
CD ≥ 3b^a^	31 (25%)	12 (29%)	19 (23%)	0.463
CD ≥ 4a^a^	20 (16%)	9 (22%)	11 (13%)	0.226
90-day CCI^b^	26.2 [17.3–42.7]	30.8 [19.95–50.85]	25.7 [14.3–39.55]	0.199
ICU stay (days)	1 [1, 2]	1 [1, 2]	1 [1, 2]	0.557
Hospital stay (days)	11 [9–22]	11 [9–28]	11 [9–20]	0.483

Values given as median [1st quartile–3rd quartile] or absolute and relative frequencies; Bold values represent significant values.

*CD* Clavien–Dindo score, *CCI* comprehensive complication index, *FFP* fresh frozen plasma, *ICU* intensive care unit, *RBC* red blood cell, *TLV* total lung volume.

^a^Refers to Dindo et al.

^b^Refers to Slankamenac.

### ROC analysis

**Figure 3:**
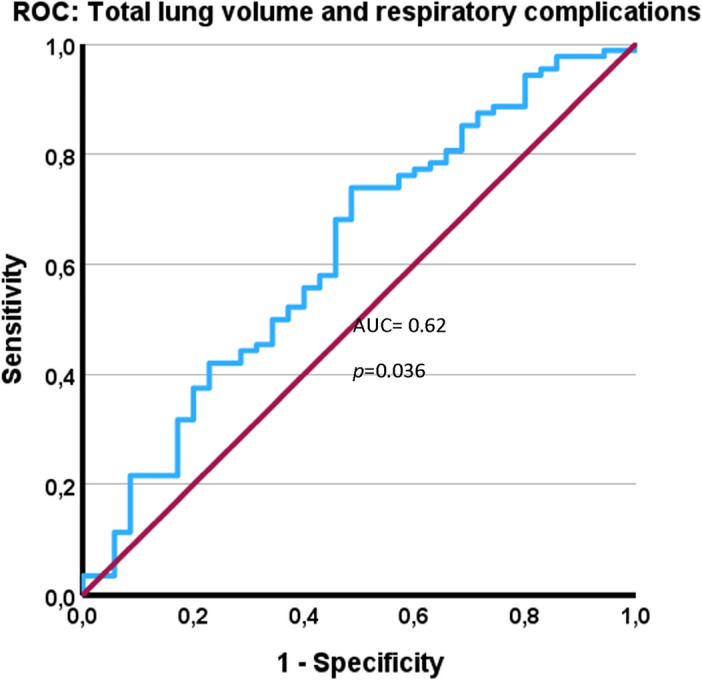
Receiver operating characteristic (ROC) curve of the main cohort: Total lung volume can predict postoperative respiratory complications. Abbreviations: AUC, Area Under the Curve.

The ROC analysis demonstrated a predictive ability for TLV with regard to respiratory complications, with an AUC of 0.62 (*p* = 0.036), as shown in Fig. 3. Youden Index analysis was used to identify the ideal cut-off value for TLV, which was determined to be 4500 cm^3^ (Youden index, YI = 0.25). The cut-off was then used to divide the cohort into two groups. The median TLV were 3883 cm^3^ and 5419 cm^3^ in the groups below and above the cut-off, respectively. More male patients (74% vs. 27%, *p* < 0.001) and a higher median height (176 cm vs. 168 cm) were observed in the TLV ≥ 4500 cm^3^ group, whereas no significant differences were seen in age or BMI. Single-step operations, staged resections and ALPPS were also equally distributed in both groups (see Table 1).

Patients with TLV < 4500 cm^3^ suffered from a two-fold increase in rate of respiratory complications (44% vs. 21%, *p* = 0.007). Furthermore, these patients also suffered from more postoperative complications in general, without achieving significance. Although not significant, the median CCI was also higher in the group with TLV < 4500cm^3^ (30.8 vs. 25.7, *p* = 0.199). The median length of ICU- and total hospital stay was similar in both groups. Further details are given in Table 2.

Univariable binary logistic regression identified TLV < 4500 cm^3^ (OR 2.992, 95%CI 1.323–6.765, *p* = 0.008), the number of intraoperative red blood cell (RBC) transfusions (OR 1.289, 95%CI 1.029–1.614, *p* = 0.027), the operating time (OR 1.004, 95%CI 1.001–1.008, *p* = 0.025) and PVE (OR 3.375, 95%CI 1.427–7.985, *p* = 0.006) as predictors for the occurrence of respiratory complications. Further predictors were right-sided hepatectomy (OR 4.000, 95%CI 1.120–14.287, *p* = 0.033), the staged resection (OR 2.421, 95%CI 1.039–5.640, *p* = 0.040) and ALPPS surgery (OR 3.429, 95%CI 1.137–10.341, *p* = 0.029). Multivariable analysis confirmed TLV < 4500 cm^3^ as an independent predictor for the occurrence of respiratory complications (OR 3.777, 95%CI 1.488–9.588, *p* = 0.005). Logistic regression results are summarized in Table 3.

**Table 3:** Univariable and multivariable binary logistic regression analysis of the whole study group, endpoint respiratory complications.

Variables	Univariable logistic regression			Multivariable logistic regression		
	OR	95% CI	*p*-value	OR	95% CI	*p*-value
TLV < 4500 cm^3^	2.992	1.323–6.765	**0.008**	3.777	1.488–9.588	**0.005**
Age	1.026	0.986–1.067	0.202			
Age ≥ 60 years	1.597	0.707–3.606	0.260			
Sex (female)	1.275	0.579–2.810	0.547			
Weight (kg)	0.988	0.966–1.011	0.304			
ASA Score^a^	0.827	0.436–1.569	0.562			
BMI	0.976	0.894–1.065	0.581			
BMI < 25	1.853	0.839–4.093	0.127			
Height	0.965	0.924–1.008	0.111			
Intraoperative RBC units	1.289	1.029–1.614	**0.027**	1.191	0.879–1.613	0.259
Intraoperative FFP units	1.112	0.992–1.247	0.070	1.013	0.851–1.206	0.882
Intraoperative platelet units	1.771	0.497–6.315	0.378			
Operating time	1.004	1.001–1.008	**0.025**	1.003	0.998–1.008	0.218
Left-sided colorectal cancer	0.994	0.785–1.257	0.958			
Synchronous liver metastases	0.630	0.244 –1.627	0.340			
Portal vein embolization (yes)	3.375	1.427 –7.985	**0.006**	2.251	0.741–6.834	0.152
Preoperative chemotherapy^b^	0.837	0.358–1.956	0.680			
Minimally invasive	0.864	0.310–2.411	0.780			
open	1.157	0.415–3.229	0.780			
anatomical	0.967	0.429–2.177	0.935			
Combined anatomical & atypical	0.397	0.139–1.138	0.086	0.561	0.176–1.794	0.330
Hemihepatectomy	0.710	0.311–1.619	0.415			
Extended hemihepatectomy/trisectionectomy	1.409	0.618–3.214	0.415			
Right-sided hepatectomy	4.000	1.120–14.287	**0.033**	2.004	0.505–7.948	0.323
Staged resection	2.421	1.039–5.640	**0.040**	0.825	0.245–2.774	0.756
ALPPS	3.429	1.137–10.341	**0.029**			

Bold values represent significant values.

*ALPPS* associating liver partition and portal vein ligation for staged hepatectomy, *ASA* American Society of Anesthesiology, *BMI* body mass index, *CI* confidence interval, *FFP* fresh frozen plasma, *RBC* red blood cell, *TLV* total lung volume.

^a^Refers to Meyer Saklad et al.

^b^Preoperative chemotherapy defined as chemotherapy given 6 months before surgery.

### Patients undergoing open surgery

The predictive ability of TLV regarding respiratory complications was stronger for patients undergoing open surgery (n = 100), with an AUC of 0.65 (*p* = 0.018), as shown in Supplemental Fig. 1. Here, a cut-off TLV value of 4494 cm^3^ (YI = 0.29) was calculated. Patients with TLV < 4494 cm^3^ suffered a 2.5-fold increase in rate of respiratory complications (48% vs. 20%, *p* = 0.004). These patients also developed more postoperative complications in general, without achieving significance (CD ≥ 3a, 61% vs. 41%, *p* = 0.055; CD ≥ 3b, 39% vs. 25%, *p* = 0.151; CD ≥ 4a: 29% vs. 13%, *p* = 0.054). The median CCI was also higher in the group with TLV < 4494 cm^3^, without achieving significance (38.1 vs. 25.7, *p* = 0.060). Although not significant, the median length of total hospital stay was also longer in the group with TLV < 4494 cm^3^ (16 vs. 11 days, *p* = 0.110), whereas the median length of ICU stay was similar in both groups. Further results are shown in Supplementary Table 1.

Univariable binary logistic regression analysis identified TLV < 4494 cm^3^ (OR 3.683, 95%CI 1.473–9.212, *p* = 0.005), the number of intraoperative RBC transfusions (OR 1.310, 95%CI 1.034–1.660, *p* = 0.025) and the number of intraoperative fresh frozen plasma (FFP) transfusions (OR 1.134, 95%CI 1.002–1.283, *p* = 0.046) as predictors for the occurrence of respiratory complications. Further predictors were confirmed in univariable binary logistic regression: operating time (OR 1.006, 95%CI 1.002–1.011, *p* = 0.006), PVE (OR 4.164, 95%CI 1.619–10.709, *p* = 0.003), staged resection (OR 2.793, 95%CI 1.114–7.005, *p* = 0.029) and ALPPS surgery (OR 4.127, 95%CI 1.284–13.260, *p* = 0.017). Multivariable analysis confirmed TLV < 4494 cm^3^ as an independent predictor (OR 4.728, 95%CI 1.374–16.274, *p* = 0.014) for the occurrence of respiratory complications. Results of the logistic regression analysis in patients with open surgery are summarized in Supplementary Table 2.

### Non-overweight patients

We investigated the prognostic capability of TLV in different BMI groups, as defined by the WHO classification, and found significant results in the non-overweight population (BMI < 25 kg/m^2^). In this combined group of normal- and underweight patients (n = 50), the ROC analysis demonstrated a good predictive ability for TLV regarding respiratory complications (AUC 0.70, *p* = 0.021), as shown in Supplemental Fig. 2. The Youden Index analysis identified 4943 cm^3^ as an optimal cut-off value in these patients (YI = 0.35). In patients with TLV < 4943 cm^3^, the rate of respiratory complications was more than double (52% vs. 20%, *p* = 0.018) and the rate of life-threatening complications (CD ≥ 4a) was more than triple (40% vs. 12%, *p* = 0.024). Although not significant, patients with TLV < 4943 cm^3^ also exhibited higher median CCI (39.7 vs. 27.6, *p* = 0.093). The median number of days on ICU (2 vs. 1, *p* = 0.127) and in hospital (19 vs. 11,* p* = 0.047) was nearly double in patients with TLV < 4943 cm^3^. Further results are shown in Supplementary Table 3.

Univariable binary logistic regression analyses identified TLV < 4943 cm^3^ (OR 4.333 95%CI 1.235–15.206, *p* = 0.022) and operating time (OR 1.006 95%CI 1.000–1011, *p* = 0.038) as predictors for the occurrence of respiratory complications in non-overweight patients. The former was also an independent predictor in the multivariable analysis (OR 6.355 95%CI 1.369–29.501, *p* = 0.018). Results are summarized in Supplementary Table 4.

### Patients 60 years and older

The predictive ability of TLV was also strong in patients 60 years or older (AUC = 0.69, *p* = 0.01), as shown in Supplementary Fig. 3. The analysis identified 5345 cm^3^ (YI = 0.35) as the optimal cut-off value in this group of 71 patients. Remarkably, patients with TLV < 5345 cm^3^ suffered from a four-fold increase in the rate of respiratory complications (44% vs. 12%, *p* = 0.004). These patients also developed more CD ≥ 3a complications (58% vs. 35%, *p* = 0.06), without achieving significance. The median CCI of these patients was significant higher (34.8 vs. 24.2, *p* = 0.007). Patient characteristics of this subgroup are given in Supplementary Table 5.

Univariable binary logistic regression analyses showed TLV < 5345 cm^3^ as a predictor for the occurrence of respiratory complications in this subgroup (OR 6.133, 95%CI 1.607–23.403, *p* = 0.008). Further predictors for the occurrence of respiratory complications were BMI < 25 kg/m^2^ (OR 2.862, 95%CI 1.017–8.055, *p* = 0.046), operating time (OR 1.005, 95%CI 1.000–1.010, *p* = 0.042) and right-sided hepatectomy (OR 6.300, 95%CI 1.319–30.091, *p* = 0.021). Height also had a significant influence on respiratory complications, with an OR of 0.937 (95%CI 0.881–0.996, *p* = 0.038), which was not found in the main study cohort. In the multivariable analysis, only TLV < 5345 cm^3^ (OR 6.225 95%CI 1.375–28.185, *p* = 0.018) was an independent predictor of respiratory complications (Supplementary Table 6).

### Patients undergoing ALPPS

Patients undergoing ALPPS (n = 15) comprised the third subgroup of our analysis and TLV was found to have the best predictive ability for respiratory complications in these patients, with AUC = 0.82 (*p* = 0.037), as shown in Supplementary Fig. 4. The cut-off value for TLV was defined as 4249 cm^3^ (YI = 0.63). All patients with TLV below the defined cut-off suffered from respiratory complications (100% vs. 30%, *p* = 0.010) and they also exhibited a significantly higher rate of life-threatening complications (CD ≥ 4a 80% vs. 30%, *p* = 0.067). The median CCI was also higher in these patients, (71.7 vs. 39.5, *p* = 0.165) without achieving significance. Further results are given in Supplementary Table 7. Logistic regression analysis was not carried out in this group, because of the limited sample size.

## Discussion

In this study, we investigated the predictive ability of preoperative CT-based 3DLV regarding postoperative respiratory complications, in patients undergoing major liver resection for CRLM. Through ROC analysis, we defined a TLV cut-off of 4500 cm^3^ and showed a two-fold higher rate of respiratory complications in patients with TLV below this value. To the best of our knowledge, this is the first study to demonstrate this in patients undergoing liver surgery.

Computerized 3DLV can be performed with relative ease on routine CT scans of the thorax. No additional CT with specific protocols is required. The repeatability of the lung volume measurements is excellent in both healthy persons, as well as patients with obstructive or restrictive lung disease, as shown by Shin et al.^22^. As previously shown^12,13^, 3DLV of routine chest CT scans allows a technically stable estimation of lung volume and is well correlated with spirometric measurements. In thoracic surgery, various studies have shown that 3DLV is able to reliably predict postoperative pulmonary function^13–16^ and to predict pulmonary complications. For example, Murakami et al. combined forced vital capacity (FVC) with TLV measurements from preoperative CT volumetry, to diagnose a lung size-function mismatch and predict cardiopulmonary complications after major lung resection for cancer ^23^. Kawakami et al. used preoperative 3DLV of emphysematous lungs to predict postoperative pulmonary complications in patients with chronic obstructive pulmonary disease undergoing resection of lung cancer^24^. Furthermore, according to Saito et al., 3DLV is able to differentiate patients who develop restrictive allograft syndrome, from those who develop bronchiolitis obliterans syndrome, after bilateral lung or heart–lung transplantation^25^.

Using 3DLV as a predictor for postoperative respiratory complications after major curative liver resection for CRLM is a novel application. We focused on patients undergoing major liver surgery, which is associated with higher rate of complications compared to minor liver resections. We found TLV to be predictive of respiratory, but not general complications in the ROC analysis (data not shown). Nevertheless, the ability to identify patients at risk of respiratory complications after major liver surgery is of great clinical value. Firstly, these complications can increase ICU- and hospital stay, as can new complications arising from their treatment (for example, pneumothorax after punction of a pleural effusion). This increases cumulative morbidity for the affected patients, as well as treatment costs. Secondly, physical status, including lung capacity, is amenable through pre-habilitation. Identification of patients at risk in the ambulatory setting provides the opportunity to improve their pulmonary status through exercise and physiotherapy. Finally, the same applies postoperatively, where intensive pulmonary therapy, physiotherapy, and regular monitoring of lung function (e.g., through clinical examination and bedside pleural sonography) may lead to prevention or earlier diagnosis of these complications.

To further investigate the predictive ability of TLV, we analyzed subgroups of interest, according to factors associated with lung volume and/or complication rates^26,27^. These included BMI < 25, age over 60, open surgery and ALPPS. Obesity is associated with reduced respiratory function and detrimental effects^28,29^. Zimmitti et al. showed that patients with BMI ≥ 30 kg/m^2^ undergoing liver surgery were more often comorbid, had longer operation duration and more intraoperative blood loss, as well as significantly higher rates of wound- and respiratory-related complications^30^. A linear correlation between BMI and TLV has been previously described by Jones et al.^26^. They found decreasing functional residual capacity (FRC) and expiratory reserve volume (ERV) with increasing BMI on all lung volume measurements. But also underweight has a negative impact on lung function, as shown by Grigsby et al. In a study with 12,396 participants, they showed individuals with lower BMI were more likely to have COPD and decreased lung function^31^. The association with lung function remained positive after excluding participants with COPD, suggesting that being underweight alone has a detrimental effect on lung function^31^. The association of BMI with lung function was apparent in our study, where the predictive ability of TLV was stronger in the non-overweight population, compared to the whole study cohort (AUC 0.70 vs. 0.62, respectively). However, we did not find a predictive ability of TLV for respiratory complications in overweight patients, probably due to the small sample size (n = 19). Further studies with larger cohorts in each BMI category are necessary to elucidate this effect.

Since lung function deteriorates from the age of 60 years onwards^32^, we analyzed this patient cohort separately. Remarkably, there was a fourfold increase in the rate of respiratory complications in patients with TLV below the defined cut-off-value for that subgroup. Reasons for increased rate of respiratory complications in older patients include a smaller lung volume and the natural effect of weakening of immune system in elderly patients, called immunosenescence^33,34^. Additionally, morphological and functional changes occur, such as smaller airway size and a decrease in lung elastic tissue, chest wall compliance, and respiratory muscle strength^35,36^. The reduced lung function in the elderly was reflected in the higher cut-off value for TLV in this subgroup, compared to the whole study population (5345 cm^3^ vs. 4500 cm^3^). This suggests that a higher lung volume is necessary to compensate for the reduced lung function.

Commonly, open liver surgery is associated with higher complications rates compared to minimal-invasive liver surgery^37^. In this cohort with patients who underwent open surgery, we found 2.5-fold higher respiratory complication rates in patients with lower TLV compared to those with higher TLV. The nearly similar cut-off value for TLV in this subgroup is explained by the fact that most patients underwent open surgery in this study (100 vs. 23).

The fourth subgroup analyzed in this study comprised patients undergoing ALPPS. The introduction of this technique was a milestone in liver surgery, allowing patients with extensive hepatic tumor burden the chance of resection, at the expense of higher rates of perioperative morbidity and mortality^21,38^. Refinements in patient selection and operative techniques resulted in significant improvements in perioperative outcomes over the years, but ALPPS remains a high-risk operation^38,39^. In our cohort, we found a significantly higher AUC value (0.82) for TLV, with regards to respiratory complications, compared to the main cohort. Additionally, all ALPPS patients below the calculated TLV cut-off suffered from respiratory complications and the rate of respiratory complications in the whole ALPSS group was twice as high as non-ALPPS patients (53% vs. 25%). This suggests a potentially great applicability of 3DLV and TLV for the prediction of respiratory complications in ALPPS patients, with the caveat of the small sample size in this study (n = 15). In any case, these results merit further investigation with a larger cohort.

Interestingly, PVE was found to be a significant risk factor for respiratory complications in the univariable logistic regression analysis. Although a quarter of our cohort (n = 31, 25%) underwent preoperative PVE, we did not analyze these patients separately, as we believe the association between PVE and respiratory complications to be indirect. In other words, these patients underwent PVE in preparation for extended or staged liver resections, which were the cause of these complications, rather than the PVE itself. This was supported by the lack of any studies in the literature reporting pulmonary complications after PVE.

Certain limitations should be considered when interpreting the results of this study, including its retrospective design, monocentric nature, and relatively small patient cohort, including subgroups. A major reason for the cohort size was the lack of thorax CT within six weeks prior to surgery, as required in the inclusion criteria of our study. We excluded older imaging, as the physical status of the patient may have significantly changed between CT and operation, especially if chemotherapy had been administered in the meantime. We did not split the dataset into training- and test-sets as this was an explorative study investigating the effects of lung volume and our goal was not to train a predictive model, rather to explore the associated effects retrospectively. The variation in timing and place of imaging between patients in our cohort is a further limiting factor. Ideally, all patients in this study would have undergone their CT on the day before surgery, at UH-RWTH, to better reflect pulmonary status at the time of operation and exclude differences in CT protocols between our center and referring hospitals or practices. Additionally, using the Brisbane classification to define major resections has inherent limitations: multiple or large atypical resections also result in significant parenchyma loss and are also associated with increased complications, due to the non-anatomical resection planes, with a larger number of transected vessels and bile ducts. Going forward, measuring the percentage of lost volume may better serve to stratify the extent of liver resection.

In any case, accounting for the study’s limitations, preoperative 3DLV with TLV measurement is a viable technique for prediction of postoperative pulmonary complications in patients undergoing major liver resection for CRLM. Particularly patients over 60 years of age, non-overweight patients, and those undergoing ALPPS could benefit from this novel application.

## Conclusion

We have shown 3DLV with TLV measurement to be a viable technique for prediction of postoperative pulmonary complications in patients undergoing major liver resection for CRLM. The methodology could be routinely applied in the preoperative setting, to identify patients at risk and commence mitigating measures, such as prehabilitation. Further studies with larger cohorts and external validation are necessary to assess and optimize this technique.

### Supplementary Information

Supplementary Information.

## Data Availability

The datasets generated during and/or analyzed during the current study are available from the corresponding author on reasonable request.

## Abbreviations

3DLV: Three-dimensional lung volumetry
ALPPS: Associating liver partition and portal vein ligation for staged hepatectomy
ASA: American society of anesthesiology
AUC: Area under the curve
BMI: Body mass index
CI: Confidence interval
CRC: Colorectal cancer
CRLM: Colorectal liver metastases
CT: Computerized tomography
CUSA: Cavitron ultrasonic surgical aspirator
CVP: Central venous pressure
DICOM: Digital imaging and communications in medicine
FVC: Forced vital capacity
ICU: Intensive care unit
IMC: Intermediate care unit
IQR: Interquartile range
KRAS: Kirsten rat sarcoma viral oncogene homolog
MRI: Magnetic resonance imaging
OR: Odds ratio
PACS: Picture archiving and communication system
PVE: Portal vein embolization
R0: Resection margin negative
R+: Resection margin positive
TLV: Total lung volume
TNM: Tumor, lymph node, metastasis
UH-RWTH: University hospital RWTH Aachen
UICC: Union for international cancer control
YI: Youden index
