# Supplementary material for: Preoperative three-dimensional lung volumetry predicts respiratory complications in patients undergoing major liver resection for colorectal metastases
Source: Sci Rep. 2024 May 8;14:10594. doi: 10.1038/s41598-024-61386-8 (PMC11079043; doi:10.1038/s41598-024-61386-8)
Supplement: Supplementary file 1 — Supplementary Information. [file 41598_2024_61386_MOESM1_ESM.docx]

**Supplement to:**

Preoperative three-dimensional lung volumetry predicts respiratory complications in patients undergoing major liver resection for colorectal metastases.

Suzan Elmaagacli^1^, Christoph Thiele^2^, Franziska Meister^1^, Philipp Menne^1^, Daniel Truhn^3^, Steven W. M. Olde Damink^4^, Johannes Bickenbach^2^, Ulf Neumann^5^, Sven Arke Lang^5^, Florian Vondran^1^, and Iakovos Amygdalos*^1^

Affiliations

^1^Department of General, Visceral, Pediatric and Transplantation Surgery, University Hospital RWTH Aachen, Aachen, Germany; ^2^Department of Operative Intensive Care and Intermediate Care, University Hospital RWTH Aachen, Aachen, Germany; ^3^Department of Diagnostic and Interventional Radiology, University Hospital RWTH Aachen, Aachen, Germany, ^4^Department of Surgery, Maastricht University Medical Center, Maastricht, The Netherlands, ^5^Department of General, Visceral, and Transplantation Surgery, University Hospital Essen, Essen, Germany;

1. Subgroup Open surgery, cut-off TLV=4494cm^3^

| Variables | All patients (n=100) | TLV < 4494cm^3^ (n=31) | TLV > 4494cm^3^ (n=69) | P-value |
| --- | --- | --- | --- | --- |
| Age (years) | 62 [56-68] | 61 [52-70] | 62 [57-68] | 0.929 |
| Sex (female) | 38 (38%) | 22 (71%) | 16 (23%) | **< 0.001** |
| BMI | 25 [23-28] | 24 [21-27] | 25 [23-28] | 0.412 |
| Height in cm | 175 [167-180] | 170 [160-174] | 176 [170-183] | **< 0.001** |
| ASA Score ^§^ | 3 [2-3] | 3 [2-3] | 3 [2-3] | 0.929 |
| ASA Score > 3 | 59 (59%) | 19 (61%) | 40 (58%) | 0.755 |
| Synchronous metastases | 72 (72%) | 23 (74%) | 49 (71%) | 0.743 |
| Right -sided colorectal cancer | 28 (28%) | 8 (26%) | 20 (29%) | 0.743 |
| Preoperative chemotherapy^#^ | N= 86  50 (58%) | N= 27  13 (48%) | N= 59  37 (63%) | 0.204 |
| Operating time (min) | 280 [235-357] | 270 [233-357] | 288 [235-358] | 0.985 |
| Intraoperative RBC units | 0 [0-2] | 0 [0-2] | 0 [0-2] | 0.877 |
| Intraoperative platelet units | 0 [0-0] | 0 [0-0] | 0 [0-0] | n/a |
| Intraoperative FFP units | 0 [0-4] | 0 [0-2] | 0 [0-4] | 0.204 |
| Extubation at ICU | 39 (39%) | 13 (42%) | 26 (38%) | 0.687 |
| Respiratory complications | 29 (29%) | 15 (48%) | 14 (20%) | **0.004** |
| CD >3a * | 47 (47%) | 19 (61%) | 28 (41%) | 0.055 |
| CD >3b * | 29 (29%) | 12 (39%) | 17 (25%) | 0.151 |
| CD >4a * | 18 (18%) | 9 (29%) | 9 (13%) | 0.054 |
| 90-day CCI ^+^ | 27.4 [18.2-45.725] | 38.1 [24.2- 64.5] | 25.7 [13.6- 41.2] | 0.060 |
| ICU stay (days) | 1 [1-2] | 1 [1-3] | 1 [1-2] | 0.143 |
| Hospital stay (days) | 11 [9-24] | 16 [9-32] | 11 [9-21] | 0.110 |

Legend: Values given as median (1st quartile – 3rd quartile) or absolute and relative frequencies; Bold values represent significant values. Abbreviations used: ASA, American Society of Anesthesiology; BMI, body mass index; CD, Clavien-Dindo score; CCI, Comprehensive Complication Index; FFP, fresh frozen plasma; ICU, intensive care unit; RBC, red blood cell; TLV, total lung volume; ^§^ refers to Meyer Saklad et al.; ^#^ Preoperative chemotherapy defined as chemotherapy given 6 months before surgery; * refers to Dindo et al. ^+^ refers to Slankamenac

1. Univariable and multivariable binary logistic regression analysis of the subgroup undergoing open surgery, endpoint respiratory complications.

| Variables | Univariable logistic regression | | | Multivariable logistic regression | | |
| --- | --- | --- | --- | --- | --- | --- |
|  | **OR** | **95% CI** | ***p*-value** | **OR** | **95% CI** | ***p*-value** |
| TLV cut-off value < 4494cm^3^ | 3.683 | 1.473 – 9.212 | **0.005** | 4.728 | 1.374 – 16.274 | **0.014** |
| Age | 1.029 | 0.983 – 1.076 | 0.217 |  |  |  |
| Age > 60 years | 1.390 | 0.566 – 3.416 | 0.473 |  |  |  |
| ASA Score ^§^ | 0.796 | 0.398 – 1.591 | 0.518 |  |  |  |
| BMI | 0.980 | 0.890 – 1.080 | 0.689 |  |  |  |
| BMI < 25 | 1.890 | 0.789 – 4.525 | 0.153 |  |  |  |
| Height | 0.956 | 0.911 – 1.004 | 0.070 | 0.972 | 0.907 -1.041 | 0.411 |
| Intraoperative RBC units | 1.310 | 1.034 – 1.660 | **0.025** | 1.061 | 0.759 – 1.482 | 0.730 |
| Intraoperative FFP units | 1.134 | 1.002 – 1.283 | **0.046** | 1.120 | 0.899 – 1.395 | 0.314 |
| Intraoperative platelet units | n/a | n/a | n/a |  |  |  |
| Operating time | 1.006 | 1.002 – 1.011 | **0.006** | 1.005 | 0.999 – 1.012 | 0.079 |
| Left-sided colorectal cancer | 0.959 | 0.746 – 1.232 | 0.743 |  |  |  |
| Synchronous liver metastases | 0.581 | 0.207 – 1.627 | 0.301 |  |  |  |
| Portal vein embolization (yes) | 4.164 | 1.619 – 10.709 | **0.003** | 3.355 | 0.855 – 13.169 | 0.083 |
| Preoperative chemotherapy^#^ | 0.884 | 0.345 – 2.263 | 0.797 |  |  |  |
| anatomical | 0.723 | 0.298 – 1.757 | 0.475 |  |  |  |
| Combined anatomical & atypical | 0.438 | 0.135 – 1.424 | 0.170 |  |  |  |
| Hemihepatectomy | 0.723 | 0.298 – 1.757 | 0.475 |  |  |  |
| Extended Hemihepatectomy/ Trisectionectomy | 1.382 | 0.569 – 3.358 | 0.475 |  |  |  |
| Right-sided Hepatectomy | 4.585 | 0.990 – 21.229 | 0.051 | 1.635 | 0.290 – 9.197 | 0.577 |
| Staged resection | 2.793 | 1.114 – 7.005 | **0.029** | 0.581 | 0.137 – 2.470 | 0.462 |
| ALPPS | 4.127 | 1.284 – 13.260 | **0.017** |  |  |  |

Legend: Bold values represent significant values. Abbreviations used: ALPPS, Associating Liver Partition and Portal vein ligation for Staged hepatectomy; ASA, American Society of Anesthesiology; BMI, body mass index; CI, confidence interval; FFP, fresh frozen plasma; RBC, red blood cell; TLV, total lung volume, ^§^ refers to Meyer Saklad et al.; ^#^ Preoperative chemotherapy defined as chemotherapy given 6 months before surgery.

1. Subgroup non-overweight patients, cut-off TLV=4943cm^3^

| Variables | All patients (n=50) | TLV < 4943cm^3^ (n=25) | TLV > 4943cm^3^ (n=25) | P-value |
| --- | --- | --- | --- | --- |
| Age (years) | 64 [57-70] | 64 [59-73] | 63 [56-70] | 0.573 |
| Sex (female) | 27 (54%) | 18 (72%) | 9 (36%) | **0.011** |
| BMI | 22 [20-23] | 22 [20-23] | 23 [21-24] | 0.377 |
| Height in cm | 170 [165-175] | 167 [161-173] | 174 [168-180] | **0.003** |
| ASA Score ^§^ | 3 [2-3] | 3 [2-3] | 3 [2-3] | 0.784 |
| ASA Score > 3 | 31 (62%) | 16 (64%) | 15 (60%) | 0.771 |
| Synchronous metastases | 39 (78%) | 20 (80%) | 19 (76%) | 0.733 |
| Right -sided colorectal cancer | 15 (30%) | 5 (20%) | 10 (40%) | 0.123 |
| Preoperative chemotherapy^#^ | N= 44  (59%) | N= 20  8 (40%) | N=24  18 (75%) | **0.019** |
| Operating time (min) | 315 [220-396] | 327 [220-431] | 315 [227-372] | 0.793 |
| Intraoperative RBC units | 0 [0-2] | 0 [0-2] | 0 [0-2] | 0.974 |
| Intraoperative platelet units | 0 [0-0] | 0 [0-0] | 0 [0-0] | 0.077 |
| Intraoperative FFP units | 0 [0-3] | 0 [0-2] | 0 [0-4] | 0.483 |
| Extubation at ICU | 22 (44%) | 10 (40%) | 12 (48%) | 0.569 |
| Respiratory complications | 18 (36%) | 13 (52%) | 5 (20%) | **0.018** |
| CD >3a * | 27 (54%) | 16 (64%) | 11 (44%) | 0.156 |
| CD >3b * | 16 (32%) | 11 (44%) | 5 (20%) | 0.069 |
| CD >4a * | 13 (26%) | 10 (40%) | 3 (12%) | **0.024** |
| 90-day CCI ^+^ | 29.85 [22.6-48.15] | 39.7 [22.6-75.05] | 27.6 [22.6-35.95] | 0.093 |
| ICU stay (days) | 1 [1-2] | 2 [1-5] | 1 [1-2] | 0.127 |
| Hospital stay (days) | 15 [10-28] | 19 [11-36] | 11 [10-21] | **0.047** |

Legend: Values given as median (1st quartile – 3rd quartile) or absolute and relative frequencies; Bold values represent significant values. Abbreviations used: ASA, American Society of Anesthesiology; BMI, body mass index; CD, Clavien-Dindo score; CCI, Comprehensive Complication Index; FFP, fresh frozen plasma; ICU, intensive care unit; RBC, red blood cell; TLV, total lung volume; ^§^ refers to Meyer Saklad et al.; ^#^ Preoperative chemotherapy defined as chemotherapy given 6 months before surgery; * refers to Dindo et al. ^+^ refers to Slankamenac

1. Univariable and multivariable binary logistic regression analysis of the subgroup with non-overweight patients, endpoint respiratory complications.

| Variables | Univariable logistic regression | | | Multivariable logistic regression | | |
| --- | --- | --- | --- | --- | --- | --- |
|  | **OR** | **95% CI** | ***p*-value** | **OR** | **95% CI** | ***p*-value** |
| TLV cut-off value < 4943cm^3^ | 4.333 | 1.235 – 15.206 | **0.022** | 6.355 | 1.369 – 29.501 | **0.018** |
| Age | 1.026 | 0.972 – 1.083 | 0.345 |  |  |  |
| Age > 60 years | 3.421 | 0.822 – 14.242 | 0.091 | 2.958 | 0.563 - 15.534 | 0.200 |
| ASA Score ^§^ | 0.525 | 0.190 – 1.450 | 0.214 |  |  |  |
| BMI | 0.973 | 0.730 – 1.298 | 0.854 |  |  |  |
| height | 0.946 | 0.881 – 1.017 | 0.133 |  |  |  |
| Intraoperative RBC units | 1.417 | 0.916 – 2.192 | 0.118 |  |  |  |
| Intraoperative FFP units | 1.124 | 0.896 – 1.410 | 0.311 |  |  |  |
| Intraoperative platelet units | 0.689 | 0.100 – 4.731 | 0.705 |  |  |  |
| Operating time | 1.006 | 1.000 – 1.011 | **0.038** | 1.007 | 0.998 – 1.015 | 0.127 |
| Left-sided colorectal cancer | 1.157 | 0.823 – 1.627 | 0.400 |  |  |  |
| Synchronous liver metastases | 0.319 | 0.061 – 1.680 | 0.178 |  |  |  |
| Portal vein embolization (yes) | 3.467 | 0.959 – 12.536 | 0.058 | 4.017 | 0.751 – 21.502 | 0.104 |
| Preoperative chemotherapy^#^ | 0.556 | 0.159 – 1.936 | 0.356 |  |  |  |
| Minimally invasive | 0.875 | 0.144 – 5.320 | 0.885 |  |  |  |
| open | 1.143 | 0.188 – 6.949 | 0.885 |  |  |  |
| anatomical | 0.684 | 0.214 – 2.188 | 0.522 |  |  |  |
| Combined anatomical & atypical | 0.511 | 0.119 – 2.200 | 0.367 |  |  |  |
| Hemihepatectomy | 1.765 | 0.531 – 5.865 | 0.354 |  |  |  |
| Extended Hemihepatectomy/ Trisectionectomy | 0.567 | 0.171 – 1.883 | 0.354 |  |  |  |
| Right-sided Hepatectomy | 3.923 | 0.433 – 35.530 | 0.224 |  |  |  |
| Staged resection | 3.000 | 0.884 – 10.184 | 0.078 | 0.577 | 0.063 – 5.271 | 0.626 |
| ALPPS | 2.700 | 0.688 – 10.602 | 0.155 |  |  |  |

Legend: Bold values represent significant values. Abbreviations used: ALPPS, Associating Liver Partition and Portal vein ligation for Staged hepatectomy; ASA, American Society of Anesthesiology; BMI, body mass index; CI, confidence interval; FFP, fresh frozen plasma; RBC, red blood cell; TLV, total lung volume, ^§^ refers to Meyer Saklad et al.; ^#^ Preoperative chemotherapy defined as chemotherapy given 6 months before surgery.

1. Subgroup age≥60 years, cut-off TLV=5345cm^3^

| Variables | All patients (n=71) | TLV < 5345cm^3^ (n=45) | TLV > 5345cm^3^ (n=26) | P-value |
| --- | --- | --- | --- | --- |
| Age (years) | 67 [63-72] | 66 [64-71] | 68 [63-74] | 0.248 |
| Sex (female) | 28 (39%) | 28 (62%) | 0 (0%) | **<0.001** |
| BMI | 25 [23-27] | 24 [21-27] | 25 [24-28] | 0.089 |
| Height in cm | 172 [166-180] | 167 [161-175] | 179 [174-183] | **<0.001** |
| ASA Score ^§^ | 3 [2-3] | 3 [2-3] | 3 [2-3] | 0.445 |
| ASA Score > 3 | 47 (66%) | 31 (69%) | 16 (62%) | 0.528 |
| Synchronous metastases | 52 (73%) | 33 (73%) | 19 (73%) | 0.981 |
| Right -sided colorectal cancer | 25 (35%) | 14 (31%) | 11 (42%) | 0.341 |
| Preoperative chemotherapy^#^ | N= 60  36 (60%) | N= 38  19 (50%) | N=22  17 (77%) | **0.038** |
| Operating time (min) | 275 [245-361] | 272 [235-359] | 301 [258-364] | 0.434 |
| Intraoperative RBC units | 0 [0-2] | 0 [0-2] | 0 [0-2] | 0.826 |
| Intraoperative platelet units | 0 [0-0] | 0 [0-0] | 0 [0-0] | 0.266 |
| Intraoperative FFP units | 0 [0-4] | 0 [0-2] | 3 [0-4] | **0.042** |
| Extubation at ICU | 31 (44%) | 19 (42%) | 12 (46%) | 0.748 |
| Respiratory complications | 23 (32%) | 20 (44%) | 3 (12%) | **0.004** |
| CD >3a * | 35 (49%) | 26 (58%) | 9 (35%) | 0.060 |
| CD >3b * | 20 (28%) | 15 (33%) | 5 (19%) | 0.203 |
| CD >4a * | 14 (20%) | 11 (24%) | 3 (12%) | 0.188 |
| 90-day CCI ^+^ | 27.2 [22.6- 44.2] | 34.8 [22.6-66.05] | 24.2 [12.2-32.375] | **0.007** |
| ICU stay (days) | 1 [1-3] | 1 [1-4] | 1 [1-2] | 0.118 |
| Hospital stay (days) | 12 [10-26] | 14 [10-34] | 11 [10-21] | 0.118 |

Legend: Values given as median (1st quartile – 3rd quartile) or absolute and relative frequencies; Bold values represent significant values. Abbreviations used: ASA, American Society of Anesthesiology; BMI, body mass index; CD, Clavien-Dindo score; CCI, Comprehensive Complication Index; FFP, fresh frozen plasma; ICU, intensive care unit; RBC, red blood cell; TLV, total lung volume; ^§^ refers to Meyer Saklad et al.; ^#^ Preoperative chemotherapy defined as chemotherapy given 6 months before surgery; * refers to Dindo et al. ^+^ refers to Slankamenac

1. Univariable and multivariable binary logistic regression analysis of the subgroup with age ≥60 years, endpoint respiratory complications.

| Variables | Univariable logistic regression | | | Multivariable logistic regression | | |
| --- | --- | --- | --- | --- | --- | --- |
|  | **OR** | **95% CI** | ***p*-value** | **OR** | **95% CI** | ***p*-value** |
| TLV cut-off value < 5345cm^3^ | 6.133 | 1.607 – 23.403 | **0.008** | 6.225 | 1.375 – 28.185 | **0.018** |
| Age | 1.049 | 0.961 – 1.143 | 0.284 |  |  |  |
| ASA Score ^§^ | 0.722 | 0.294 – 1.773 | 0.477 |  |  |  |
| BMI | 0.911 | 0.795 – 1.045 | 0.182 |  |  |  |
| BMI < 25 | 2.862 | 1.017 – 8.055 | **0.046** | 1.684 | 0.518 – 5.478 | 0.386 |
| height | 0.937 | 0.881 – 0.996 | **0.038** |  |  |  |
| Intraoperative RBC units | 1.122 | 0.920 – 1.369 | 0.255 |  |  |  |
| Intraoperative FFP units | 0.951 | 0.799 – 1.132 | 0.574 |  |  |  |
| Intraoperative platelet units | 0.776 | 0.116 – 5.199 | 0.794 |  |  |  |
| Operating time | 1.005 | 1.000 – 1.010 | **0.042** | 1.005 | 0.999 – 1.010 | 0.091 |
| Left-sided colorectal cancer | 1.062 | 0.810 – 1.391 | 0.664 |  |  |  |
| Synchronous liver metastases | 0.463 | 0.134 – 1.599 | 0.223 |  |  |  |
| Portal vein embolization (yes) | 2.786 | 0.920 – 8.432 | 0.070 | 1.229 | 0.335 – 4.517 | 0.756 |
| Preoperative chemotherapy^#^ | 0.394 | 0.131 – 1.186 | 0.097 |  |  |  |
| Minimally invasive | 1.233 | 0.322 – 4.726 | 0.760 |  |  |  |
| open | 0.811 | 0.212 – 3.108 | 0.760 |  |  |  |
| anatomical | 0.852 | 0.297 – 2.443 | 0.766 |  |  |  |
| Combined anatomical & atypical | 0.505 | 0.126 – 2.021 | 0.334 |  |  |  |
| Hemihepatectomy | 0.433 | 0.151 – 1.241 | 0.119 |  |  |  |
| Extended Hemihepatectomy/ Trisectionectomy | 2.308 | 0.806 – 6.607 | 0.119 |  |  |  |
| Right-sided Hepatectomy | 6.300 | 1.319 – 30.091 | **0.021** | 3.265 | 0.586 – 18.189 | 0.177 |
| Staged resection | 2.443 | 0.822 – 7.260 | 0.108 |  |  |  |
| ALPPS | 2.389 | 0.615 – 9.272 | 0.208 |  |  |  |

Legend: Bold values represent significant values. Abbreviations used: ALPPS, Associating Liver Partition and Portal vein ligation for Staged hepatectomy; ASA, American Society of Anesthesiology; BMI, body mass index; CI, confidence interval; FFP, fresh frozen plasma; RBC, red blood cell; TLV, total lung volume, ^§^ refers to Meyer Saklad et al.; ^#^ Preoperative chemotherapy defined as chemotherapy given 6 months before surgery.

1. Subgroup undergoing ALPPS, cut-off TLV=4249cm^3^

| Variables | All patients (n=15) | TLV < 4249cm^3^ (n=5) | TLV > 4249cm^3^ (n=10) | P-value |
| --- | --- | --- | --- | --- |
| Age (years) | 63 [53-71] | 73 [53-80] | 61 [52-65] | 0.129 |
| Age > 60 years | 10 (66.7%) | 4 (80%) | 6 (60%) | 0.439 |
| Sex ratio (F/M) | 8 (53.3%)/ 7 (46.7%) | 4 (80%) | 4 (40%) | 0.143 |
| BMI | 23 [20-25] | 20 [19-23] | 24 [21-26] | 0.165 |
| Height in cm | 173 [160-176] | 163 [158-171] | 175 [170-179] | 0.129 |
| ASA Score ^§^ | 3 [3-3] | 3 [2-3] | 3 [3-3] | 0.371 |
| ASA Score > 3 | 12 (80%) | 3 (60%) | 9 (90%) | 0.171 |
| Synchronous vs. metachronous liver metastases | 12 (80%) vs. 3 (20%) | 4 (80%) vs. 1 (20%) | 8 (80%) vs. 2 (20%) | 1 |
| Right- vs. left-sided colorectal cancer | 6 (40 %) vs. 9 (60%) | 1 (20%) vs. 4 (80%) | 5 (50%) vs 50%) | 0.264 |
| Preoperative chemotherapy^#^ | 12 (80%) | 4 (80%) | 8 (80%) | 1 |
| Surgery duration (min) | 357 [278-450] | 357 [278-357] | 400 [235-470] | 1 |
| Intraoperative RBC units | 2 [0-4] | 2 [1-5] | 2 [0-4] | 0.953 |
| Intraoperative platelet units | 0 [0-0] | 0 [0-0] | 0 [0-0] | 0.768 |
| Intraoperative FFP units | 3 [0-6] | 4 [1-5] | 3 [0-11] | 1 |
| Extubation at ICU | 4 (26.7%) | 2 (40%) | 2 (20%) | 0.409 |
| Respiratory complications | 8 (53.3%) | 5 (100%) | 3 (30%) | **0.010** |
| CD >3a * | 12 (80%) | 5 (100%) | 7 (70%) | 0.171 |
| CD >3b * | 10 (66.7%) | 4 (80%) | 6 (60%) | 0.439 |
| CD >4a * | 7 (46.7%) | 4 (80%) | 3 (30%) | 0.067 |
| 90-day CCI ^+^ | 54.5 [34.8-73.1] | 71.7 [50.9-86.55] | 39.5 [25.3-72.925] | 0.165 |
| ICU stay (days) | 3 [2-7] | 3 [2-11] | 3 [1-6] | 0.440 |
| Hospital stay (days) | 31 [24-65] | 31 [23-52] | 33 [22-76] | 0.859 |

Legend: Values given as median (1st quartile – 3rd quartile) or absolute and relative frequencies; Bold values represent significant values. Abbreviations used: ASA, American Society of Anesthesiology; BMI, body mass index; CD, Clavien-Dindo score; CCI, Comprehensive Complication Index; FFP, fresh frozen plasma; ICU, intensive care unit; RBC, red blood cell; TLV, total lung volume; ^§^ refers to Meyer Saklad et al.; ^#^ Preoperative chemotherapy defined as chemotherapy given 6 months before surgery; * refers to Dindo et al. ^+^ refers to Slankamenac


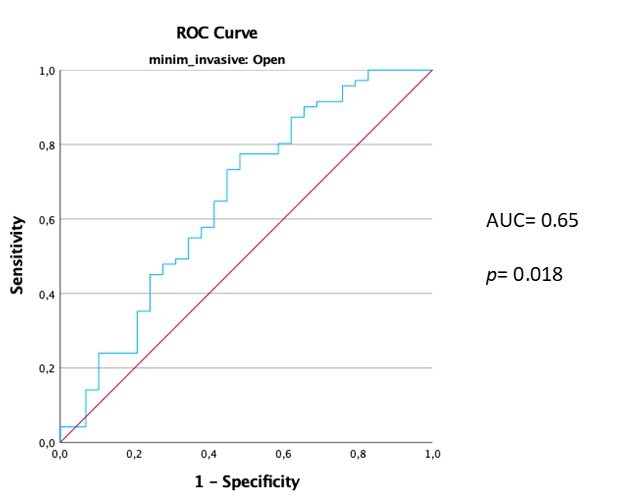


1. Receiver operating characteristic (ROC) curve of the subgroup Open surgery: Total lung volume can predict postoperative respiratory complications. Abbreviations: AUC, Area Under the Curve


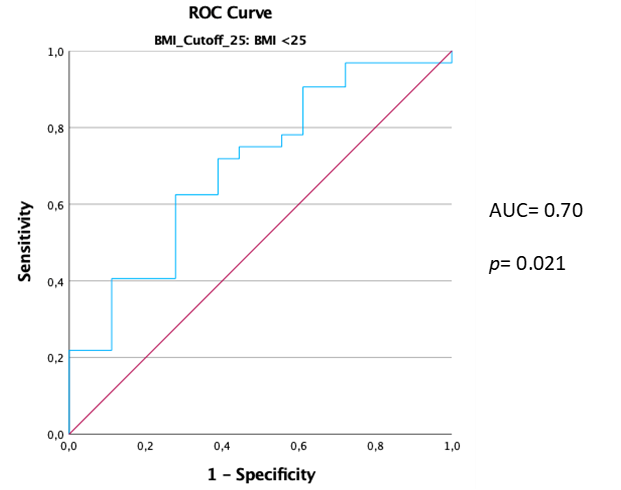


1. Receiver operating characteristic (ROC) curve of the subgroup Non-overweight patients: Total lung volume can predict postoperative respiratory complications. Abbreviations: AUC, Area Under the Curve


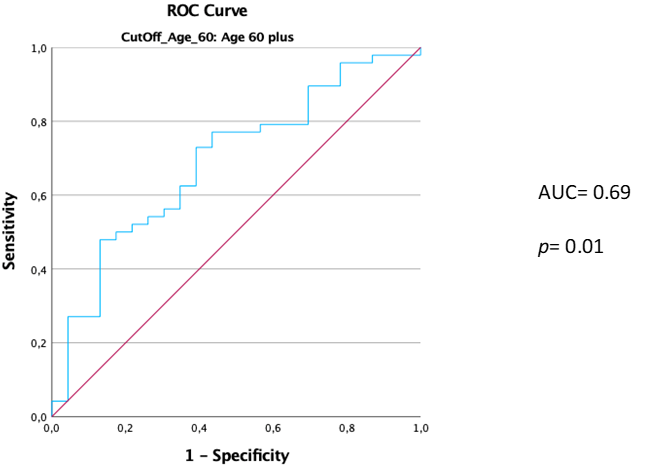


1. Receiver operating characteristic (ROC) curve of the subgroup age >60 years: Total lung volume can predict postoperative respiratory complications. Abbreviations: AUC, Area Under the Curve

**
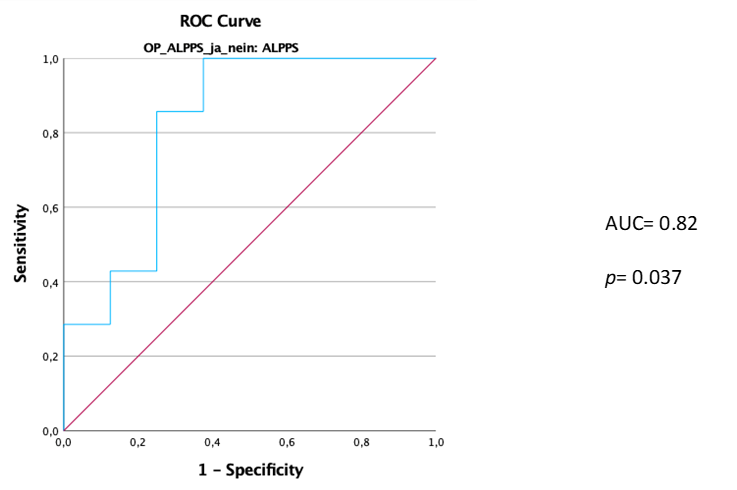
**

1. Receiver operating characteristic (ROC) curve of the subgroup ALPPS: Total lung volume can predict postoperative respiratory complications. Abbreviations: AUC, Area Under the Curve
